# Assessment of body composition as a predictor of disease severity in patients with COVID-19

**DOI:** 10.36416/1806-3756/e20240414

**Published:** 2025-06-20

**Authors:** Patrícia Blau Margosian Conti, Mauro Alexandre Pascoa, Maria Ângela Gonçalves de Oliveira Ribeiro, Carla Cristina Souza Gomez, Aline Priscila de Souza, Tayná Castilho, Gil Guerra, Daniela Souza Paiva Borgli, Eulalia Sakano, Silvana Dalge Severino, José Dirceu Ribeiro, UNICOVIDS Study Group*

**Affiliations:** 1. Laboratório de Fisiologia Pulmonar - LaFiP - Centro de Investigação em Pediatria - CIPED - Faculdade de Ciências Médicas - FCM - Universidade Estadual de Campinas - Unicamp - Campinas (SP) Brasil.; 2. Laboratório de Crescimento e Desenvolvimento - LabCreD - Centro de Investigação em Pediatria - CIPED - Faculdade de Ciências Médicas - FCM - Universidade Estadual de Campinas - Unicamp - Campinas (SP) Brasil.

**Keywords:** COVID-19, Body composition, Risk factors, Quality of life

## Abstract

**Objective:**

This study sought to estimate body composition values by disease severity in patients recovered from COVID-19.

**Methods:**

This was an observational, analytical, prospective cross-sectional study involving patients recovered from COVID-19 and employing the following assessment methods: bioelectrical impedance analysis, dual-energy X-ray absorptiometry, and air displacement plethysmography.

**Results:**

A total of 210 volunteers were included. Demographic characteristics, comorbidities, and body composition values highlighted significant differences between men and women across disease severity levels.

**Conclusions:**

Sex differences influence the severity of COVID-19. Our results provide a cross-sectional analysis of the impact of body composition on COVID-19 patients, showing risk factors and parameters that can contribute to the treatment, health recovery, and quality of life of this population.

## INTRODUCTION

Patients who have recovered from COVID-19 continue to suffer from sequelae and treatments that impact their quality of life and health restoration.^1^ Anthropometric characteristics play a role in the severity of disease in COVID-19 patients. Obesity has been identified as a characteristic feature of the disease,^2^ whereas the musculoskeletal system plays a protective role in regulating the immune system.^3^ In these two aspects, the influence of body composition on the severity of COVID-19 has yet to be better understood, leaving knowledge gaps regarding prevention and treatment strategies for COVID-19. 

COVID-19 is a severe acute respiratory syndrome caused by SARS-CoV-2.^4^ It is an infection that affects the immune system through the pulmonary airways,^5^ leading to a rapid systemic viral spread affecting the respiratory, cardiovascular, and nervous systems.^6^ ^,^ ^7^ The symptoms of COVID-19 can range from mild to critical,^4^ often requiring ICU admission, with high mortality rates^8^ and sequelae, including changes in body composition.^9^

Body composition analysis has been shown to be relatively accurate^10^ when employed in health assessment and in identifying risk factors in a clinical setting.^11^ Fat mass and fat-free mass are well-established markers in the literature.^12^ Although the use of various integrated devices involving different methods and procedures presents challenges,^13^ ^,^ ^14^ they were rigorously controlled across all evaluation procedures in the present study. 

The primary objective of the present study was to estimate body composition values by disease severity in patients recovered from COVID-19. Secondary objectives included a comparison of body composition assessments, including bioelectrical impedance analysis (BIA), dual-energy X-ray absorptiometry (DXA), and air displacement plethysmography (ADP). 

## METHODS

The present study was approved by the Research Ethics Committee of the *Universidade Estadual de Campinas*, located in the city of Campinas, Brazil (Protocol no. CAAE 36305120.0.0000.5404). This was an observational, analytical, prospective cross-sectional study examining the relationship between COVID-19 severity and body composition measurements. The study sample consisted of patients recovered from COVID-19 and selected from various sources, including hospital records, health centers, local authorities, and personal referrals. The time elapsed between SARS-CoV-2 infection (confirmed by RT-PCR) or hospitalization and body composition assessment ranged from 90 days to 12 months. Inclusion criteria were as follows: having a positive laboratory or clinical test for COVID-19; being > 18 years of age; and having no preexisting chronic diseases. 

Patients were instructed to wear light clothing, with no metals or objects that could interfere with the assessments. They were also instructed as to what they were allowed to eat before the assessments. Access to the testing environment was restricted to authorized personnel. The testing environment comprised a waiting room, an individual screening room, and adequately equipped assessment rooms. The temperature was maintained at 21°C, being constantly monitored with digital and mercury thermometers, with an atmospheric pressure of 937 hPa.^15^ All patients underwent an initial screening, in which physiological markers were measured. Clinical history taking included colds, fever, blood pressure changes, and frailty, as well as questions regarding the decision to withdraw from the study. All participating patients gave written informed consent. 

Body composition assessments included the following: body weight (in kg), measured to the nearest 0.1 kg with a digital scale (PL-200; Filizola S.A., São Paulo, Brazil)^16^; height (in cm), measured to the nearest 0.1 cm with a wall-mounted stadiometer (Holtain Ltd, Crosswell, UK)^17^; BIA with a 450 bioimpedance analyzer (Biodynamics Corporation, Seattle, WA, USA)^19^ ^,^ ^20^; DXA with a Lunar iDXA device (GE Healthcare Technologies, Inc., Chicago, IL, USA)^21^; and ADP with a Bod Pod GS body composition tracking system (COSMED srl, Rome, Italy).^22^ Upon completing the assessments, all participating patients had a debriefing interview with the principal investigator and received a participation certificate showing the main results of the study. 

Body composition assessment utilized nine variables of interest as independent predictors: BMI; resting metabolic rate (RMR), in kcal/day; body volume (BV), in L; body density (BD); body surface area (BSA), in cm^2^; total body fat (TBF), in kg; fat-free mass (FFM), in kg; relative skeletal muscle index (RSMI); and visceral fat mass (VFM), in kg. The dependent variable was disease severity (nonsevere, severe, or critical), in accordance with the 2020 WHO criteria.^23^ Study biases were addressed by taking into consideration age, self-reported physical activity level,^24^ ^,^ ^25^ comorbidities, and other factors identified during the initial screening. 

Patient data records on the devices included sex, age, body mass, height, self-reported ethnicity, and physical activity level as references for body composition calculations, as well as data cross-referencing between devices. Bioimpedance measures, body imaging measures, and air displacement measures formed both device-specific equations and combined equations. 

The results of the assessments were recorded in a Microsoft Excel spreadsheet or Windows Notepad file and then transferred to the IBM SPSS Statistics software package, version 25.0 (IBM Corporation, Armonk, NY, USA). Descriptive analyses verified data dispersion and normality. Categorical variables determined the distribution among groups, and continuous variables were analyzed through comparisons. Measures with statistically significant values (p < 0.05) were further analyzed for explanatory analysis between groups. 

## RESULTS

A total of 210 volunteers were included in the present study. Corrected post hoc tests found an effect size of f² = 0.28 and a power 1-β = 0.80 (α = 0.05), calculated using G*Power software, version 3.1.9.7 (Institute for Experimental Psychology, Dusseldorf, Germany). Of the 210 study participants, 110 (52.4%) were women. Of the 89 patients who had had nonsevere COVID-19, 60 (67.4%) were women. Of the 67 patients who had had critical COVID-19, 44 (65.7%) were men. Of the 54 patients who had had severe COVID-19, 27 (50%) were women and 27 (50%) were men. 

Categorical tests found differences in COVID-19 severity only for the sex variable [X²(2) = 16.942; p < 0.001]. Among men and women, differences were observed between those with nonsevere disease and those with critical disease (−3.7 vs. 3.6 and 3.7 vs. −3.6, respectively). 

Comparative tests between sexes by disease severity level showed significant differences (p < 0.05), the exception being VFM. To avoid type II errors, all variables were considered separately by sex. Continuous variables demonstrated normality for both sexes, as assessed by the Shapiro-Wilk test. Among men, significant results were found for body weight, BMI, RMR, BSA, TBF, FFM, and VFM (p < 0.001), as well as for RSMI (p = 0.001). Among women, significant results were found for body weight (p = 0.001), BMI (p = 0.005), RMR (p = 0.010), BSA (p = 0.025), TBF (p = 0.012), FFM (p = 0.006), and VFM (p = 0.008). Dispersion values for men are shown in Table 1. Dispersion values for women are shown in Table 2. The time elapsed between COVID-19 diagnosis and body composition assessment was adjusted to prevent it from acting as a confounding factor. After the adjustment, the Kruskal-Wallis test was performed, and no significant differences were observed. 

**Table 1:** Measures of dispersion for male patients recovering from COVID-19, by disease severity.

Parameter		Nonsevere COVID-19		Severe COVID-19		Critical COVID-19	Men		
	N	mean ± SD*/median (IQR)**	N	mean ± SD*/median (IQR)**	N	mean ± SD*/median (IQR)**	Nonsevere vs. severe	Nonsevere vs. critical	Severe vs. critical
Age, years*	29	48.4 ± 12.2	27	53.8 ± 12.6	44	50.3 ± 9.6			
Body weight, kg**	29	81.6 (18.7)	27	82.9 (33.9)	44	94.6 (23.5)	0.872	0.014	0.326
Height, cm*	29	176.2 ± 6.2	27	172.9 ± 8.8	44	174.9 ± 7.5			
PCR-days**	29	246 (309)	27	173 (225)	44	332 (332)			
BMI**	29	26.9 (5.0)	27	28.8 (8.2)	44	31.2 (4.2)	0.085	p < 0.001	0.520
RMR**	29	1,721.4 (286.0)	27	1,692.6 (571.7)	44	1,933.8 (342.3)			
BV**	29	77.2 (19.3)	24	81.1 (37.9)	42	90.4 (27.1)	0.314	0.029	1.000
BD*	29	1.041 ± 0.020	24	1.023 ± 0.018	42	1.025 ± 0.016	0.002	0.001	1.000
TGV**	29	4.1 (0.5)	24	4.1 (0.8)	42	4.0 (0.5)			
BSA**	29	197.4 (22.2)	24	196.7 (48.6)	42	211.3 (29.0)			
TBF**	29	23.1 (10.5)	27	27.1 (18.7)	44	32.4 (10.3)	0.037	< 0.001	0.699
FFM**	29	56.2 (8.1)	27	54.7 (12.4)	44	58.5 (12.2)			
RSMI**	29	8,6 (1,5)	27	8,5 (2,0)	44	9,0 (1,5)			
VFM**	29	2,108.9 (1,494.0)	27	2,917.9 (2,186.7)	44	3,432.9 (1,482.3)	0.030	< 0.001	0.859

PCR-days: time elapsed between SARS-CoV-2 infection (confirmed by RT-PCR) and body composition assessment; RMR: resting metabolic rate; BV: body volume; BD: body density; TGV: total gas volume; BSA: body surface area; TBF: total body fat; FFM: fat-free mass; RSMI: relative skeletal muscle index; and VFM: visceral fat mass. *ANOVA. **Kruskal-Wallis test.

**Table 2:** Measures of dispersion for female patients recovering from COVID-19, by disease severity.

Parameter		nonsevere COVID-19		severe COVID-19		critical COVID-19	Women		
	N	mean ± SD*/median (IQR)**	N	mean ± SD*/median (IQR)**	N	mean ± SD* / median (IQR)**	nonsevere vs. severe	nonsevere vs. critical	severe vs. critical
Age, years*	60	42.8 ± 11.8	27	48.0 ± 10.9	23	55.5 ± 10.6	0.15	< 0.001	0.068
Body weight, kg**	60	74.2 (21.4)	27	86.0 (24.1)	23	84.1 (23.3)	0.003	0.023	1.000
Height, cm*	60	161.9 ± 6.9	27	160.5 ± 6.2	23	159.9 ± 7.0			
PCR-days**	60	198 (161)	27	303 (332)	23	320 (256)			
BMI**	60	27.6 (8.3)	27	32.7 (7.7)	23	33.1 (7.0)	0.001	0.002	1.000
RMR**	60	1,630.7 (188.0)	27	1,746.4 (243.9)	23	1,704.7 (229.6)	0.007	0.083	1.000
BV**	60	67.1 (28.7)	27	78.6 (26.3)	23	84.3 (25.4	0.124	0.014	1.000
BD*	60	1.015 ± 0.020	27	1.000 ± 0.014	23	0.997 ± 0.012	0.001	< 0.001	1.000
TGV**	60	3.1 (0.3)	27	3.1 (0.3)	23	3.2 (0.4)			
BSA**	60	178.0 (20.7)	27	188.5 (20.9)	23	185.3 (25.3)	0.014	0.182	1.000
TBF**	60	31.6 (15.3)	27	39.5 (20.9)	23	38.0 (10.7)	0.005	0.009	1.000
FFM**	60	40.2 (4.9)	27	42.9 (7.6)	23	42.4 (11.5)	0.013	0.432	0.848
RSMI**	60	7,0 (1,0)	27	7,8 (1,6)	23	7,6 (1,8)	0.005	0.264	0.820
VFM**	60	2,484.5 (1,550.3)	27	3,444.1 (2,214.6)	23	3,122.3 (1,461.6)	0.003	0.009	1.000

PCR-days: time elapsed between SARS-CoV-2 infection (confirmed by RT-PCR) and body composition assessment; RMR: resting metabolic rate; BV: body volume; BD: body density; TGV: total gas volume; BSA: body surface area; TBF: total body fat; FFM: fat-free mass; RSMI: relative skeletal muscle index; and VFM: visceral fat mass. *ANOVA. **Kruskal-Wallis test.

Anthropometric comparisons distinguished men from women among severity groups. Men had lower RSMI, whereas women had normal RSMI. The mean age was 69.4 ± 4.1 years for men and 69.3 ± 2.9 years for women. In the group of patients with nonsevere disease, women were younger (42.8 ± 11.8 years vs. 48.4 ± 12.2 years; p = 0.042) and lighter (74.2 ± 21.4 kg vs. 81.6 ± 18.7 kg; p = 0.005), although men were taller (176.2 ± 6.2 cm vs. 161.9 ± 6.9 cm; p < 0.001). 

In the group of patients with severe disease, men were taller (172.9 ± 8.8 cm vs. 160.5 ± 6.2 cm; p < 0.001) and had a lower BMI (28.8 ± 8.2 vs. 32.7 ± 7.7; p = 0.025). In the group of patients with critical disease, men were younger (50.3 ± 9.6 years vs. 55.5 ± 10.6 years; p = 0.046), heavier (94.6 ± 23.5 kg vs. 84.1 ± 23.3 kg; p = 0.023), and taller (174.9 ± 7.5 cm vs. 159.9 ± 7.0 cm; p < 0.001). With regard to disease severity in men, differences were observed between those with nonsevere disease and those with severe disease regarding BD (p = 0.002), TBF (p = 0.037), and VFM (p = 0.030), as well as between those with nonsevere disease and those with critical disease regarding body weight (p = 0.014), BMI (p < 0.001), BV (p = 0.029), BD (p = 0.001), TBF (p < 0.001), and VFM (p < 0.001). 

With regard to disease severity in women, differences were observed between those with nonsevere disease and those with severe disease regarding body weight (p = 0.003), BMI (p=0.001), RMR (p=0.007), BV (p=0.124), BD (p = 0.001), BSA (p = 0.014), TBF (p = 0.005), FFM (p = 0.013), RSMI (p = 0.005), and VFM (p = 0.003), as well as between those with nonsevere disease and those with critical disease regarding age (p<0.001), body weight (p = 0.023), BMI (p = 0.002), BV (p = 0.014), BD (p < 0.001), TBF (p = 0.009), and VFM (p = 0.009). No differences were found between men or women with severe disease and men or women with critical disease. 

## DISCUSSION

The present study allowed a precise comparison of body composition between male and female post-COVID-19 patients.^11^ The novelty of the present study lies in its integrating different devices and body composition markers, all of which were controlled at the same time of assessment. This allowed us to include a large number of post-COVID-19 patients, ranging from those who were asymptomatic to those who were still recovering in terms of health and quality of life. 

Our study revealed a trend across disease severity levels, with the most significant differences found between nonsevere and critical cases. Age and male sex were found to be predictors of disease severity.^26^ Risk factors such as ethnicity, BMI, and cardiometabolic comorbidities have been analyzed in hospitalized patients.^27^ No differences were found regarding ethnicity, whereas sex was a criterion for grouping in most markers. Anthropometry showed greater predictive power for women, including comparative age and better frequency outcomes among the groups. The literature indicates that men may be more vulnerable than women in terms of immunological, hormonal, and genetic health. However, reduced access and lack of personal care are also risk factors.^28^ It is known that women in the post-recovery period are more vulnerable to experiencing a lower quality of life.^29^

Height and body weight were used as demographic markers for calculating BMI, being widely employed in health contexts.^26^ Interestingly, BMI, originally known as the Quetelet index,^30^ has limitations and is often erroneously associated with weight, localized fat deposits, and even social aspects or aspects related to physical activity and age.^31^

When we compared BMI by sex, we found that only the group of patients with severe COVID-19 showed differences. Body weight, which is an important modulator, did not follow this pattern. Height, on the other hand, influenced all three groups of disease severity. When we analyzed the results by disease severity, we found that BMI correlated with most markers, with a greater influence on women. 

Among body components, fat mass, represented by VFM, showed a strong association with BMI^26^ and BD.^32^ In our study, these body components showed a strong relationship among groups for both men and women. Only BMI differed from the main results in men. 

FFM had no influence on severity in men and showed inconsistent differences in women. These findings suggest an imbalance in body composition, with obesity (BMI > 30 kg/m^2^)^26^ ^,^ ^32^ standing out as one of the main characteristics of COVID-19,^2^ increasing the risk of severe disease. It is known that a balanced relationship between adequate levels of adipose tissue and lean mass constitutes an important health protective factor.^33^

Obesity, independently of COVID-19, is an inflammatory disorder that exacerbates health problems, influencing the immune and neuroendocrine systems.^34^ Under the effect of COVID-19, obesity can impair respiratory function through hormonal signaling and the release of proinflammatory adipocytokines from adipose tissue itself.^35^ Conversely, musculoskeletal tissue acts as the main endocrine organ, regulating the immune system.^3^ Through physical activity, it produces anti-inflammatory myokines capable of reducing the systemic concentration of inflammatory cytokines.^36^ This mechanism directly links exercise to muscle mass volume.^37^ The relative musculoskeletal index, composed of the sum of appendicular musculature, is an important DXA marker based on body height.^38^ Its results correlated with lean components, showing no influence on men and limited influence on women. The values found were low for health in men, and women showed a normal index. 

In the present study, age also included patients with immunosenescence. From a longevity perspective, it is known that a decrease in muscle and bone tissue, with a peak in adipose tissue increase, occurs on average between the ages of 65 and 70 years, which suggests a significant risk factor extended to the general population.^39^ The decline in immune function accompanies the decrease in lean mass in the physiological process of immunosenescence.^3^ The importance of these changes suggests that the musculoskeletal tissue is the main organ in health prevention in response to advancing age. In this physiological process, there is a decrease in immunity to new infections, as well as in the effects of vaccination, along with an increase in chronic systemic inflammation.^40^

Sex differences influence the severity of COVID-19. The use of various assessment tools allowed us to obtain precise values in the distribution of groups. Adiposity indices and FFM can be modulated as health protective factors. Our results support outpatient evaluations for appropriate interventions, including nutritional guidance and structured physical activity programs. 

The present study allowed a cross-sectional analysis of the impact of body composition on COVID-19 patients. Specifically, the identified markers may assist in comparing COVID-19 with other comorbidities and physiological parameters. 
